# Frailty in Older Adults with Heart Failure

**DOI:** 10.1007/s11897-026-00785-6

**Published:** 2026-10-31

**Authors:** Miroslava Valentová

**Affiliations:** 1 Department of Geriatrics, University Medical Center Göttingen, Robert-Koch-Str. 42, 37075 Göttingen, Germany; 2 German Center for Cardiovascular Research (DZHK), Göttingen site, Germany

**Keywords:** Heart failure, Frailty, Aging, Myokines, Geroscience, Geriatric assessment

## Abstract

**Purpose of Review:**

Heart failure (HF) and frailty are increasingly prevalent with population aging, frequently coexist, and share pathophysiological mechanisms. This review summarizes recent evidence on shared biological pathways and management strategies in patients with HF and frailty.

**Recent Findings:**

Emerging data suggest that accumulation of damaged cellular components, mitochondrial dysfunction, cellular senescence, and chronic inflammation contribute to both conditions. Altered myokine-mediated signaling further highlights skeletal muscle as an important peripheral link between HF and frailty and as a potential therapeutic target. Frailty is increasingly recognized as a clinically relevant measure and potentially modifiable treatment target. Current evidence, largely derived from small or single-center studies, suggests that individualized multidomain interventions, including exercise training, nutritional optimization, and geriatric co-management, may improve patient-centered outcomes.

**Summary:**

Management should integrate guideline-directed HF therapy with targeted treatment of geriatric syndromes, guided by multidimensional assessment and individual patient goals. Multicenter trials in frail HF populations are needed to inform evidence-based management recommendations.

## Introduction

Population aging is contributing to a growing number of older adults affected by heart failure (HF) and frailty, both of which increase in prevalence with advancing age and frequently coexist. The prevalence of HF exceeds 10% among individuals aged 80 years and older in many populations [1]. In this age group, heart failure with preserved ejection fraction (HFpEF) is the predominant phenotype and a major cause of hospitalization and mortality [2, 3]. HF is also associated with loss of independence, greater need for home care services, and a greater risk of nursing home admission [4]. These outcomes are particularly relevant because many older patients prioritize quality of life, functional independence, and remaining at home over longevity alone [5, 6].

Older adults with HF frequently have multiple geriatric conditions, including frailty, sarcopenia, malnutrition, gait impairment, and cognitive impairment. These conditions rarely occur in isolation and may aggravate HF symptoms, impair self-care and treatment adherence, and increase the complexity of clinical management. In a recent study of older ambulatory patients with HFpEF, nearly half had at least three geriatric impairments spanning physical, cognitive, and psychosocial domains [7].

Among these conditions, frailty is particularly important because it provides an integrated measure of a patient’s vulnerability. Frailty is a multidimensional and dynamic state of reduced physiological reserve in which relatively minor stressors, such as an infection, medication change, or hospitalization, may precipitate disproportionate and prolonged functional decline. It is distinct from chronological age: an older person may remain robust, whereas frailty may also occur in younger patients with advanced HF. Frailty affects approximately 45% of patients with HF, although prevalence estimates vary according to patient age, clinical setting, and the assessment instrument used [8].

Frailty is clinically important because it is consistently associated with mortality, hospitalization, functional decline, and loss of independence in patients with cardiac disease [9]. In the OPTIMISE HFpEF cohort, frailty was associated with a more than fourfold higher risk of the one-year composite endpoint of all-cause hospitalization or death [7]. Its prognostic relevance extends beyond multimorbidity: in older surgical patients, frailty predicted longer hospital stay, delirium, postoperative morbidity, and one-year mortality, whereas multimorbidity was associated only with postoperative morbidity [10]. Frailty may also increase susceptibility to adverse events during HF management and periprocedural complications [11–14]. Conversely, HF may promote or accelerate frailty through congestion, malnutrition, inflammation, impaired skeletal muscle perfusion, and physical inactivity. Frailty should therefore not be interpreted as a reason to withhold evidence-based HF treatment. Rather, it identifies patients who may require additional supportive measures, including closer monitoring, individualized initiation and titration of therapy, and multidomain interventions addressing physical function, nutrition, cognition, medication burden, and social support.

Given its high prevalence, prognostic relevance, and potential modifiability, routine frailty screening should be considered in all patients with HF aged ≥ 70 years, irrespective of ejection fraction or care setting. Numerous frailty instruments are available. Among them, the Clinical Frailty Scale (CFS) is well validated, widely used across clinical settings, and can be completed within minutes [15]. Evidence from older surgical patients indicates that the risk of adverse postoperative outcomes already increases at a CFS score of 4, corresponding to prefrailty and preceding the conventional frailty threshold of CFS ≥ 5 [10]. A positive screening result should prompt a multidimensional geriatric assessment and an individualized management plan integrating guideline-directed HF therapy with rehabilitation, nutritional support, medication review, cognitive assessment, and social care.

This review summarizes the clinical and pathophysiological interplay between HF and frailty and discusses management considerations for older patients. These include pharmacological and interventional HF therapies, multidomain strategies targeting modifiable components of frailty, and the integration of individualized goals and patient-centered outcomes into cardiovascular care.

## Shared Mechanisms of Heart Failure and Frailty: A Geroscience Perspective

Conventional cardiometabolic risk factors and clinical conditions remain major drivers of incident HF. In 8,558 participants from the PREVEND cohort followed for 25 years, hypertension, hypercholesterolemia, obesity, smoking, atrial fibrillation, chronic kidney disease, myocardial infarction, and type 2 diabetes collectively accounted for approximately 46–71% of incident HF cases, depending on sex and HF phenotype. Hypertension was strongly associated with both HFrEF and HFpEF, whereas hypercholesterolemia contributed more prominently to HFrEF and obesity to HFpEF [16].

Despite their different proximal effects, these risk factors converge on common pathways of cellular stress, tissue injury, and maladaptive remodeling. Several aging-related mechanisms have been implicated in HF and appear particularly relevant in HFpEF. These include impaired macroautophagy and mitophagy, dysregulated proteostasis, mitochondrial dysfunction, cellular senescence, immunosenescence, chronic low-grade inflammation, and clonal hematopoiesis [17]. The strength of evidence differs between individual pathways and ranges from experimental models to analyses of human tissue and circulating biomarkers. An overview of these mechanisms, selected molecular targets, and their potential clinical implications is provided in Table 1.

**Table 1:** Selected cellular and molecular mechanisms linking heart failure, aging, and frailty

Mechanism	Background	Key molecular pathways/targets and investigational therapeutic agents	Evidence in HFpEF	Evidence in aging / frailty
Impaired autophagy and mitophagy	**Autophagy** is a lysosome-dependent recycling pathway that removes long-lived proteins, aggregates, and damaged organelles; **mitophagy** selectively clears damaged mitochondria. Impairment at initiation, autophagosome formation, fusion, or lysosomal degradation reduces autophagic flux [18, 19].	**Key molecular pathways/targets:** AMPK-mTORC1-ULK1; Beclin 1-VPS34; ATG5/7; LC3-II and p62/SQSTM1; TFEB/LAMP2; PINK1-Parkin and BNIP3/NIX; NAD+-insulin/IGF-1 signaling. **Investigational agents:** nicotinamide, rapamycin, spermidine, schaftoside.	**Preclinical (rodent models):** Nicotinamide restored cardiac autophagy and mitophagy and improved remodeling and diastolic dysfunction; these benefits were lost after cardiomyocyte-specific Atg5 deletion [20] **Preclinical (aged mice):** In aged AngII-infused mice, schaftoside restored lysosomal autophagic flux through allosteric inhibition of CaMKII-delta and attenuated remodeling and diastolic dysfunction [21]	**Preclinical aging/sarcopenia:** In old mice, sustained Hand2 expression in sympathetic neurons preserved sympathetic and neuromuscular innervation, increased skeletal-muscle autophagy markers, and attenuated muscle atrophy and loss of force [22] **Preclinical cardiac aging:** Aged mouse hearts showed reduced autophagic flux; restoring GSK-3β-ULK1 signaling attenuated hypertrophy, fibrosis, dysfunction, and senescence [23].
Mitochondrial dysfunction and redox imbalance	Mitochondria produce ATP and coordinate substrate utilization, calcium handling, and stress signaling. Mitochondrial dysfunction reduces energetic reserve and may increase reactive oxygen species, inflammation, and cell injury [24].	**Key molecular pathways/targets:** Electron-transport chain; cardiolipin; PGC-1α; NAD+-sirtuins; NRF2; fission/fusion proteins DRP1, MFN1/2, and OPA1. **Investigational agents:** CoQ10; elamipretide; mitochondria-targeted antioxidants.	**Clinical (observational):** Myocardial biopsies from patients with HFpEF showed mitochondrial fragmentation and reduced mitochondrial area [25]. **Clinical (interventional):** The Q-SYMBIO trial suggested a potential benefit of CoQ10 supplementation in chronic HF; however, the study was relatively small and was conducted before the widespread implementation of contemporary foundational medical therapy [26]. **Clinical (interventional):** Elamipretide, a mitochondria-targeted peptide designed to enhance mitochondrial function, did not improve left ventricular function [27].	**Clinical (observational):** Pre-frail older adults had slower muscle phosphocreatine recovery and lower abundance and activity of respiratory-complex than fit controls [28].
Inflammaging and immune dysregulation	**Inflammaging** is chronic low-grade inflammation that rises with age. **Immunosenescence** is age-related remodeling of innate and adaptive immunity, combining reduced immune competence with chronic inflammatory activation.	**Key molecular pathways/targets:** NLRP3; IL-1β, IL-6, TNF-α, and CRP; cGAS-STING and Toll-like receptors; macrophage polarization; T-cell exhaustion. **Investigational approach:** IL-1/NLRP3 blockade.	**Clinical (observational):** In patients with HFpEF, circulating IL-6 levels have been associated with greater symptom burden, reduced exercise capacity, and increased upper-body adiposity [29]. **Clinical (interventional):** In the post-MI CANTOS population, IL-1β inhibition with canakinumab was associated with fewer HF hospitalizations. However, HF was neither the primary study indication nor a primary endpoint; therefore, these findings do not establish canakinumab as an HF treatment [30].	**Clinical (observational):** Higher circulating levels of inflammatory biomarkers, including IL-6, CRP, IL-1RA, YKL-40, and elastase, have been associated with frailty [31].
Cellular senescence and the SASP	Senescent cells show persistent stress signaling and stable cell-cycle arrest; cardiomyocytes can develop a senescence-like phenotype. Their secretory phenotype (SASP) can promote inflammation and fibrosis. **Senolytics:** selectively remove senescent cells. **Senomorphs:** suppress deleterious senescence-associated signaling without inducing senescent-cell clearance.	**Key molecular pathways/targets:** p16INK4a-Rb; p53-p21; gamma-H2AX; SA-β-gal; SASP mediators IL-6, IL-8, TGF-β, and matrix metalloproteinases. **Senolytics:** dasatinib plus quercetin, fisetin, navitoclax. **Senomorphs:** mTOR/SASP modulation.	**Clinical (observational):** Circulating senescent leukocytes have been associated with HFpEF severity [32]. **Preclinical:** Navitoclax reduced senescent cells and improved LVEF and remodeling in angiotensin-II-induced HF in mice [33].	**Clinical (observational):** Senescent cell populations were identified in skeletal muscle biopsies from older and frail adults [34]. **Preclinical:** Transfer of senescent cells caused physical dysfunction; intermittent dasatinib plus quercetin improved physical function and survival in old mice [35].
Age-related clonal hematopoiesis of indeterminate potential (CHIP)	A hematopoietic stem cell acquires a somatic driver mutation and expands without overt hematologic malignancy. Mutant myeloid cells may amplify inflammation and cardiovascular injury.	**Key molecular pathways/targets:** DNMT3A, TET2, ASXL1, JAK2, TP53, PPM1D; variant allele fraction; TET2-NLRP3-IL-1β and JAK2-JAK/STAT signaling. **Investigational approach:** No CHIP-directed HF therapy has been established.	**Clinical (observational):** TET2-mediated CHIP has been described in patients with HFpEF and may be associated with diastolic dysfunction and worse clinical outcomes [36]. **Preclinical:** TET2-deficiency worsened ischemic and pressure-overload HF in mice [37].	**Clinical (observational):** CHIP prevalence increases markedly with age and is associated with higher all-cause mortality and cardiovascular risk [38].

*AMPK* AMP-activated protein kinase; *Ang II* angiotensin II; *ASXL1* additional sex combs-like 1; *ATG5/ATG7* autophagy-related proteins 5/7; *ATP* adenosine triphosphate; *BNIP3* BCL2-interacting protein 3; *CaMKII-δ* calcium/calmodulin-dependent protein kinase II delta; *CANTOS* Canakinumab Anti-inflammatory Thrombosis Outcomes Study; *cGAS-STING* cyclic GMP-AMP synthase-stimulator of interferon genes; *CHIP* clonal hematopoiesis of indeterminate potential; *CoQ10* coenzyme Q10; *CRP* C-reactive protein; *DNMT3A* DNA methyltransferase 3 α; *DRP1* dynamin-related protein 1; *GSK-3β* glycogen synthase kinase 3 β; *HAND2/Hand2* heart and neural crest derivatives expressed 2; *HF* heart failure; *HFpEF* heart failure with preserved ejection fraction; *IGF-1* insulin-like growth factor 1; *IL* interleukin; *IL-1RA* interleukin-1 receptor antagonist; *JAK/STAT* Janus kinase/signal transducer and activator of transcription; *LAMP2* lysosome-associated membrane protein 2; *LC3-II* lipidated microtubule-associated protein 1 light chain 3; *LV* left ventricular; *LVEF* left ventricular ejection fraction; *MFN* mitofusin; *MI* myocardial infarction; *mTOR* mechanistic target of rapamycin; mTORC1 mechanistic target of rapamycin complex 1; *NAD+* nicotinamide adenine dinucleotide; *NIX* NIP3-like protein X; *NLRP3* NOD-, LRR-, and pyrin domain-containing protein 3; *NRF2* nuclear factor erythroid 2-related factor 2; *OPA1* optic atrophy 1; *p16INK4a* cyclin-dependent kinase inhibitor 2A; *p21* cyclin-dependent kinase inhibitor 1A; *p53* tumor protein p53*; p62/SQSTM1* sequestosome 1; *PGC-1α* peroxisome proliferator-activated receptor-γ coactivator 1α; *PINK1* PTEN-induced kinase 1; *PPM1D* protein phosphatase Mg2+/Mn2+-dependent 1D; *Q-SYMBIO* Coenzyme Q10 as Adjunctive Treatment of Chronic Heart Failure trial; *Rb* retinoblastoma protein; *ROS* reactive oxygen species; *SA-β-gal* senescence-associated β-galactosidase; *SASP* senescence-associated secretory phenotype; *TFEB* transcription factor EB; *TGF-β* transforming growth factor β; *TNF-α* tumor necrosis factor α; *TP53* tumor protein p53; *ULK1* unc-51-like autophagy-activating kinase 1; *VPS34* vacuolar protein sorting 34; *YKL-40* chitinase-3-like protein 1; *γ-H2AX* phosphorylated histone H2AX

Biological aging affects many of these regulatory systems, creating a vulnerable substrate in which additional cardiovascular stressors, such as hypertension, are less effectively accommodated. Consequently, cardiovascular risk factors and aging may reinforce each other, promoting both HF and frailty.

A central feature shared by HF, aging, and frailty is the progressive accumulation of damaged proteins and organelles resulting from an imbalance between cellular damage and its repair or removal. Dysfunction of macroautophagy and the ubiquitin–proteasome system has been implicated in this process [17]. Macroautophagy is a lysosome-dependent recycling pathway that removes long-lived proteins, protein aggregates, and larger cellular components, including damaged organelles [18]. By contrast, the ubiquitin–proteasome system primarily degrades individual short-lived, regulatory, misfolded, or damaged proteins. Although functionally distinct, these systems interact through ubiquitin-dependent substrate recognition and adaptor proteins such as p62/SQSTM1 [39]. However, autophagic and proteasomal activity is not uniformly reduced during aging and may vary by tissue and biological context. Indeed, preserved or enhanced expression of autophagy-related genes has been associated with healthy aging and longevity [40]. Thus, aging-related disease is better understood as involving dysregulated proteostasis, which ultimately promotes the accumulation of damaged proteins and organelles.

Reduced damage clearance promotes persistent cellular stress and cellular senescence. Senescent cells remain metabolically active but exhibit stable cell-cycle arrest or, in post-mitotic cardiomyocytes, a senescence-like phenotype. Through the senescence-associated secretory phenotype (SASP), these cells release pro-inflammatory cytokines, chemokines, growth factors, and matrix-remodeling proteins that can promote inflammation, fibrosis, and dysfunction in neighboring cells. Cellular senescence therefore contributes to “inflammaging”, a low-grade inflammation associated with aging [32].

This biological overlap between HF and frailty provides the rationale for applying a geroscience framework to HF. Geroscience investigates whether targeting fundamental mechanisms of aging can delay age-related diseases and preserve physiological resilience. Experimental approaches relevant to HF include NAD⁺ repletion, modulation of insulin/IGF-1 signaling to restore autophagy, senolytic therapies, and inhibition of inflammatory pathways such as NLRP3–interleukin-1β signaling [20, 26, 37]. However, most evidence remains preclinical. Translation is limited by the pleiotropic and context-dependent functions of aging pathways, insufficient biomarkers of target engagement, incomplete modeling of multimorbidity and frailty, and potential adverse effects of chronically modifying essential homeostatic processes [17].

Among established HF interventions, exercise training and intravenous iron provide the strongest human evidence for modulating senescence-related signatures and improving skeletal muscle mitochondrial function, respectively, although senescence data were derived mainly from older adults without HF [41, 42]. Evidence for sacubitril/valsartan enhancing autophagy, mitophagy, and mitochondrial function is limited to circulating mononuclear cells [43]. Preclinical data suggest that sodium–glucose cotransporter 2 (SGLT2) inhibitors may enhance autophagy through AMPK–mTORC1 signaling and reduce senescent-cell burden [44, 45], while GLP-1/GIP-based therapies reduce systemic inflammation in obesity-related HFpEF [46, 47]. Whether these aging-related mechanisms substantially contribute to the clinical benefits of established HF therapies in humans remains uncertain.

### Myokine Signaling in the Muscle–Heart–Brain Axis

The cellular mechanisms of aging described above are not confined to the myocardium. Both HF and aging are systemic conditions in which alterations in skeletal muscle structure, function, and metabolism contribute substantially to reduced physiological reserve. Functional impairment in older patients with HF is therefore not solely explained by central cardiac limitations. In several studies, skeletal muscle mass and quality were more closely associated with peak oxygen consumption than conventional measures of cardiac function, particularly left ventricular ejection fraction [48, 49]. Conversely, exercise training improves peak oxygen consumption in patients with HF largely through peripheral adaptations in skeletal muscle rather than improvements in cardiac function [50].

Skeletal muscle is therefore not only a determinant of mobility and exercise capacity, but also a key peripheral organ in the HF–frailty continuum. Accounting for approximately 30–40% of total body mass, it consists predominantly of slow-twitch type I and fast-twitch type II myofibers embedded in a specialized microenvironment of muscle stem cells, vasculature, immune cells, Schwann cells, and motor axons. Disruption of this system with aging and chronic disease contributes to sarcopenia, a progressive loss of muscle strength, mass, and function that may occur as a primary age-related condition or secondary to chronic diseases such as HF [51].

Skeletal muscle biopsies from patients with chronic HF have revealed a shift from oxidative type I toward more glycolytic type II fibers, reduced mitochondrial density and oxidative capacity, impaired capillarization, and increased inflammatory signaling, oxidative stress, proteolysis, and apoptosis [52]. Some of these abnormalities overlap with skeletal muscle aging, which is characterized by motor-unit remodeling and denervation, mitochondrial dysfunction, altered proteostasis, and chronic low-grade inflammation [51, 53]. However, the patterns are not identical: healthy aging is particularly associated with type II fiber atrophy, whereas HF more commonly involves a relative shift from type I to type II fibers [51]. The coexistence of HF and aging may therefore further reduce skeletal muscle reserve and accentuate functional impairment.

An important question is whether skeletal muscle dysfunction merely reflects advanced HF or actively contributes to its progression. One promising, although insufficiently explored, mechanism involves altered myokine signaling. Beyond its role in locomotion, skeletal muscle functions as an endocrine organ. Myokines are cytokines, peptides, and other signaling molecules produced by skeletal muscle, many of which are released or regulated in response to contraction. They exert autocrine and paracrine effects within muscle and may act endocrinologically on distant organs. Through these pathways, myokines can influence energy metabolism, inflammation, endothelial function, cardiac remodeling and contractility, as well as neuroplasticity and cognition. This interorgan communication provides the conceptual basis for a muscle–heart–brain axis [54].

In aging and HF, physical inactivity and muscle wasting may alter myokine production and promote maladaptive signaling. This may contribute to a vicious cycle of sarcopenia, frailty, worsening exercise intolerance, progressive HF symptoms, and cognitive vulnerability. However, circulating myokine concentrations should be interpreted cautiously because many proposed myokines are produced by multiple tissues and are influenced by renal clearance, adiposity, inflammation, and medication use.

Among the most extensively studied myokines with potential relevance to HF and aging are irisin, brain-derived neurotrophic factor (BDNF), apelin, interleukin-6 (IL-6), and myostatin (Table 2). Irisin has been implicated in exercise-induced metabolic adaptation, mitochondrial function, and neurocognitive signaling [55–58]. BDNF is produced predominantly in the central nervous system; skeletal muscle is a secondary source, and muscle-derived BDNF appears to act mainly through local autocrine or paracrine pathways [59–63]. Apelin may promote angiogenesis, mitochondrial biogenesis, and cardiac function, although it is produced by several tissues and is not muscle-specific [64–67]. The effects of IL-6 are context-dependent: transient muscle-derived IL-6 released during exercise may support metabolic adaptation, whereas persistently elevated circulating IL-6 is associated with systemic inflammation and adverse outcomes [68, 69]. By contrast, myostatin inhibits muscle growth and may contribute to sarcopenia and frailty. However, higher serum myostatin levels were associated with a higher plasma amyloid-β42/40 ratio in an observational cohort of older adults, a biomarker pattern consistent with lower cerebral amyloid burden and suggesting a potential neuroprotective association of myostatin [70–73]. Overall, altered myokine signaling provides a plausible framework linking skeletal muscle dysfunction to cardiac and cognitive vulnerability in older patients with HF, although its causal relevance and therapeutic modifiability in humans remain uncertain.

**Table 2:** Selected myokines in heart failure and aging

Myokine	Background and main sources	Key biological actions	Heart failure evidence	Aging/frailty evidence	References
Predominantly decreased in HF/frailty (context-dependent)					
Irisin (FNDC5)	• FNDC5-derived, exercise-responsive peptide • Mainly skeletal muscle; also heart and adipose tissue	• ↑ mitochondrial function and glucose/lipid metabolism • ↓ cardiac hypertrophy, fibrosis and apoptosis (preclinical) • ↑ myogenesis (autocrine)	• Human: ↓ in chronic HFrEF and cachexia/sarcopenia • Animal: ↑ irisin → ↓ hypertrophy/fibrosis • No direct HF-specific cognition evidence	• Human sarcopenia cohorts: ↕ • Animal: ↓ muscle/metabolic decline • AD mice: ↑ hippocampal plasticity/memory	[55–58]
BDNF	• Neurotrophin; main source: CNS (hippocampus/cortex) • Secondary source: muscle (mainly pro-BDNF)	• ↑ neuronal survival/plasticity • ↑ muscle lipid oxidation • ↑ myocardial repair (preclinical)	• Human: ↓ BDNF; associated with ↓ exercise capacity and worse prognosis • Animal/cell: ↑ survival/repair signaling • HF with cognitive impairment: neuronal exosomal BDNF ↓ (small study)	• Human: ↓ in frailty • Exercise: transient ↑; response ↕ • ↑ BDNF associated with ↑ learning, memory and executive function	[59–63]
Apelin	• Peptide ligand of APLNR/APJ • Sources: muscle, heart, endothelium and adipose tissue	• ↑ muscle regeneration, autophagy and mitochondrial biogenesis • ↑ cardiac contractility and angiogenesis; ↓ fibrosis • ↑ arterial vasodilation	• Human: apelin ↕; APLNR/APJ ↓ in advanced HFrEF • Animal: ↑ contractility; ↓ hypertrophy/fibrosis • No direct HF-specific cognition evidence	• Human/rodent: apelin ↓ with age • Restoration: ↑ muscle function, autophagy and mitochondrial biogenesis • Rodent neurodegeneration models: ↓ neuroinflammation; ↑ memory • Human cognitive evidence lacking	[64–67]
Musclin (OSTN)	• Natriuretic peptide-like myokine • Sources: mainly skeletal muscle; also bone	• ↑ endurance and mitochondrial biogenesis • ↑ CNP signaling/contractility; ↓ fibroblast activation	• Human: OSTN ↓ in cachectic HF muscle • Mouse: deletion worsens HF; overexpression ↓ dysfunction/fibrosis • No direct HF-specific cognition evidence	• Direct aging/frailty evidence limited • Mouse activity studies: ↑ musclin → ↑ endurance • No established cognition evidence	[74–76]
Predominantly increased or stress-responsive in HF/frailty					
Myostatin (GDF8)	• TGF-β family ligand; ActRII-SMAD2/3 signaling • Sources: mainly skeletal muscle; also myocardium	• ↓ muscle growth and protein synthesis • ↑ proteolysis, atrophy and cardiac fibrosis	• Tissue: ↑ in HF muscle/heart; exercise training ↓ • Circulating levels: ↕ • Animal: blockade ↓ wasting/cardiac injury • No direct HF-specific cognition evidence	• Human: ↕ by sex, assay and lean mass • Blockade: ↑ lean mass; functional benefit ↕ • Older adults: ↑ myostatin associated with ↓ amyloid burden; causality unclear	[70–73]

Abbreviations: *AD* Alzheimer’s disease, *ActRII*, activin type II receptor, *APLNR/APJ,* apelin receptor, *BDNF* brain-derived neurotrophic factor, *CNP* C-type natriuretic peptide, *CNS* central nervous system, *FNDC5* fibronectin type III domain-containing protein 5, *GDF8* growth differentiation factor 8, *HF* heart failure, *HFrEF* heart failure with reduced ejection fraction, *OSTN* osteocrin gene, *pro-BDNF* BDNF precursor, *SMAD2/3* SMAD family members 2 and 3, *TGF-β*, transforming growth factor beta.

## Recent Developments in the Management of Patients With HF and Frailty

Current HF guidelines do not provide separate therapeutic recommendations based solely on chronological age, reflecting the fact that age alone does not adequately capture physiological reserve. By contrast, frailty is increasingly recognized as clinically relevant by cardiology societies [77]. The Heart Failure Association of the European Society of Cardiology considers frailty assessment crucial in patients with HF and advocates a multidimensional approach encompassing clinical, functional, psycho-cognitive, and social domains [78]. The AHA/ACC/HFSA guideline recognizes frailty as a potential barrier to effective HF self-care and identifies frailty assessment as a relevant component of HF management [79].

### Patient-Centered Goals and Frailty as a Treatment Target

Defining appropriate treatment goals and outcome measures is a key step in the management of patients with HF and frailty. Older patients may prioritize independence, self-care capacity, quality of life, and remaining at home over prolongation of life alone [6, 80]. Conventional endpoints such as mortality and hospitalization should therefore be complemented by standardized measures of activities of daily living, autonomy, quality of life, and days alive and out of hospital [81–83]. Harmonization of assessment instruments would further improve comparability and evidence synthesis across studies.

A major barrier to evidence-based care is the underrepresentation of frail patients in large randomized HF trials. Because frailty is rarely measured directly, evidence is often inferred from age-based subgroup analyses. However, chronological age is not a reliable surrogate for frailty. Older trial populations often include relatively fit individuals, whereas mobility limitations, cognitive impairment, and multimorbidity may limit participation of frail patients. Consequently, findings from age-based post hoc analyses may have limited applicability to this population. Future HF trials should systematically assess and report frailty, ensure adequate representation of frail individuals, or specifically target patients with HF and frailty.

Importantly, frailty should not only be considered a marker of risk, but also a potentially modifiable treatment target. This supports the use of systematic frailty assessment and, where appropriate, its use as a prespecified secondary or exploratory outcome in clinical studies [84]. Because frailty reflects deficits across multiple domains, its management requires interventions beyond conventional HF therapy. Consistent with this, current HF guidelines recommend individualized, multimodal management for patients with HF and frailty [77, 79]. The selection of treatment components should be guided by a comprehensive geriatric assessment or a comparable multidimensional assessment adapted to the care setting. Such assessments may identify modifiable deficits in physical function, nutrition, cognition, medication burden, mood, comorbidity burden, and social support, thereby informing individualized interventions such as exercise training, nutritional support, medication review, management of cognitive or depressive symptoms, and optimization of social care.

### Geriatric Co-Management and Multidomain Interventions

Geriatric rehabilitation programs are well suited to perform comprehensive geriatric assessment and deliver individualized multidomain interventions. However, the available evidence remains largely derived from small, single-center studies, as highlighted by a recent systematic review of geriatric care in 3,531 older patients with cardiac disease [85].

In acute hospital care, early geriatric co-management in 309 older patients hospitalized with cardiac disease reduced complications, including delirium, constipation, and infections, and was associated with less decline in activities of daily living, whereas effects on readmission and mortality were not clearly demonstrated [86]. Importantly, geriatric involvement does not appear to prolong hospitalization; in a subsequent study, it was associated with an approximately one-day shorter length of stay [87].

Post-discharge cardiology–geriatric co-management may reduce subsequent health-care utilization. In SENECOR, joint cardiology–geriatric follow-up reduced 12-month all-cause rehospitalization among patients aged ≥ 75 years, although no significant effects were observed on HF-related hospitalization or mortality [88]. By contrast, another single-center study reported fewer HF rehospitalizations, lower all-cause mortality, and more days alive and out of hospital within 12 months after early post-discharge co-management [89].

Stronger evidence exists for selected components of geriatric care. In REHAB-HF, an early, individualized 12-week rehabilitation program targeting strength, balance, mobility, and endurance improved lower-extremity physical function, assessed by the Short Physical Performance Battery (SPPB), in predominantly frail or pre-frail patients aged ≥ 60 years hospitalized for acute decompensated HF. The greatest benefit was observed in more frail patients. However, the intervention did not significantly reduce six-month rehospitalization [90].

Nutritional interventions may benefit selected patients with HF and documented malnutrition. In PICNIC, individualized six-month nutritional support reduced the composite of all-cause mortality or HF readmission in malnourished patients hospitalized for acute HF, although the small sample size and early termination limit generalizability [91].

Ongoing multicenter randomized trials, including REHAB-HFpEF and CHART, are expected to further clarify the role of multidomain rehabilitation in older patients with HFpEF [92, 93].

### Initiation and Titration of Foundational Medical Therapy (FMT) in Patients with HF and Frailty

In the absence of specific contraindications, FMT according to HF phenotype should not be withheld solely because of chronological age or frailty. Yet frail patients remain substantially undertreated. In the prospective FLAGSHIP cohort of 1,041 patients with HFrEF, use of triple therapy with an ACE inhibitor or ARB, beta-blocker, and mineralocorticoid receptor antagonist declined from 40.2% in the least frail to 23.4% in the most frail patients, and treatment with fewer drug classes was associated with worse outcomes across frailty categories [94].

Trial analyses suggest that frailty does not attenuate the relative efficacy of HF therapies. Dapagliflozin reduced clinical events across frailty categories in DAPA-HF and DELIVER, with greater absolute benefit in the most frail patients due to higher baseline risk [84]. Similar consistency has been reported for finerenone in FINEARTS-HF [95]. These findings indicate that frail patients may derive comparable relative, and potentially greater absolute, benefit from effective therapy.

Concerns about adverse treatment effects commonly hinder initiation or uptitration of FMT in frail patients. Although frailty is associated with a higher overall adverse-event burden, randomized subgroup analyses suggest that treatment-related excess risk compared with placebo is not substantially increased by age or frailty [96–98]. However, these analyses may be underpowered because frail patients are often underrepresented. Real-world AGING-HF data reported more frequent urinary and genital infections among patients receiving SGLT2 inhibitors than among those not treated with SGLT2 inhibitors [99]. Thus, frail patients require closer monitoring during FMT initiation and titration. At the same time, adverse events should not automatically be attributed to HF medication; temporal association, temporary dose reduction or withdrawal, and cautious rechallenge may help clarify causality.

Another debated issue in frail patients with HF is the pace of FMT uptitration and the relevance of target doses. STRONG-HF showed that rapid initiation and uptitration of renin–angiotensin system inhibitors, beta-blockers, and mineralocorticoid receptor antagonists within two weeks reduced the 180-day risk of HF readmission or all-cause death. However, the trial population was relatively young, with a mean age of 63 years and did not formally assess frailty [100]. Although no significant treatment-by-age interaction was observed, the limited number of very old patients restricts conclusions for frail geriatric populations.

In clinical practice, rapid uptitration may conflict with the geriatric principle of “start low, go slow”. In frail patients, lower starting doses and individualized target doses may be appropriate, particularly in the context of altered pharmacokinetics and pharmacodynamics. In CHARM, older patients derived similar benefit from candesartan despite receiving a somewhat lower mean daily dose [96]. Thus, FMT should be initiated without unnecessary delay, but uptitrated at an individualized pace, guided by close monitoring and tolerability rather than rigid adherence to target doses.

## Conclusion

Frailty is emerging as one of the most clinically relevant geriatric syndromes in older patients with HF and is the strongest predictor of adverse outcomes among geriatric conditions. Its importance is particularly evident in HFpEF, where frailty and HF share several features of pathological aging. Future HF care should incorporate routine frailty screening in all older patients with HF. In patients with prefrailty or frailty, assessment should identify modifiable deficits across mobility, muscle health, nutrition, cognition, mood, medication burden, and social support. These domains should then guide targeted interventions alongside guideline-directed HF therapy. Importantly, available data suggest that frail patients may derive substantial absolute benefit from evidence-based HF treatment, while several HF therapies may also modulate aging-related mechanisms. High-quality trials deliberately enrolling frail patients are needed to define optimal treatment intensity, multidomain interventions, and patient-centered outcomes for this growing population.

## Key References

- Goyal P, Zainul O, Sharma Y, Reich A, Osma P, Lau JD, et al. Geriatric Vulnerabilities Among Adults With Heart Failure With Preserved Ejection Fraction. JACC Adv. 2025;4:101602.
  - ○ This study highlights the high burden and prognostic relevance of geriatric vulnerabilities in older adults with HFpEF and supports routine multidimensional assessment in this population.
- Castro C, Delwarde C, Shi Y, Roh J. Geroscience in heart failure: the search for therapeutic targets in the shared pathobiology of human aging and heart failure. J Cardiovasc Aging. 2025;5:10.20517/jca.2024.15.
  - ○ This review provides the geroscience framework for understanding shared biological mechanisms between aging and HF, including proteostasis, senescence, inflammation, mitochondrial dysfunction, and clonal hematopoiesis.
- Ramírez-Vélez R, González A, García-Hermoso A, Amézqueta IL, Izquierdo M, Díez J. Revisiting skeletal myopathy and exercise training in heart failure: Emerging role of myokines. Metabolism. 2023;138:155348.
  - ○ This review summarizes skeletal muscle abnormalities in HF and discusses myokines as potential mediators of muscle-heart crosstalk and exercise-related benefits
- Raijmann RCMA, van Dalen TN, Koek HL, van der Meer MG, Emmelot-Vonk MH, Keijsers CJPW. Effect of geriatric care on health outcomes in older patients with cardiac disease: A systematic review. Arch Gerontol Geriatr. 2025;132:105786.
  - ○ This systematic review summarizes available evidence for geriatric care in older cardiac patients and highlights potential benefits for complications and quality of life, while emphasizing the limited quality of current evidence.
- Butt JH, Jhund PS, Belohlávek J, de Boer RA, Chiang C-E, Desai AS, et al. Efficacy and Safety of Dapagliflozin According to Frailty in Patients With Heart Failure: A Prespecified Analysis of the DELIVER Trial. Circulation. 2022;146:1210–24.
  - ○ This prespecified analysis shows that dapagliflozin benefits were consistent across frailty categories in patients with HFmrEF or HFpEF, with greater absolute benefit among more frail patients due to higher baseline risk.

## Data Availability

No datasets were generated or analysed during the current study.

## References

[1] Sayed A, Vasan RS, Harrell FE, Butler J. Trends in the Prevalence, Associated Risk Factors, and Health Burden of Heart Failure in the United States, 1988 to 2023. JACC Am Coll Cardiol Foundation. 2025;86:2542–64. 10.1016/j.jacc.2025.09.1503. 41295936

[2] Hamada T, Kubo T, Kawai K, Nakaoka Y, Yabe T, Furuno T, et al. Clinical characteristics and frailty status in heart failure with preserved vs. reduced ejection fraction. ESC Heart Fail. 2022;9:1853–63. 10.1002/ehf2.13885. PMC9065850 35355441

[3] Bozkurt B, Ahmad T, Alexander KM, Baker WL, Bosak K, Breathett K, et al. Heart Failure Epidemiology and Outcomes Statistics: A Report of the Heart Failure Society of America. J Card Fail. 2023;29:1412–51. 10.1016/j.cardfail.2023.07.006.

[4] Rørth R, Fosbøl EL, Kragholm K, Mogensen UM, Jhund PS, Petrie MC, et al. Initiation of domiciliary care and nursing home admission following first hospitalization of heart failure patients: a nationwide cohort study. Clin Epidemiol. 2018;10:917–30. 10.2147/CLEP.S164795. PMC6080879 30123004

[5] Gure TR, Kabeto MU, Blaum CS, Langa KM. Degree of Disability and Patterns of Caregiving among Older Americans with Congestive Heart Failure. J Gen Intern Med. 2008;23:70–6. 10.1007/s11606-007-0456-1. PMC2173919 18030537

[6] Krishnan S, Gogoi M, Tarrant C, Conroy S, Clayton L, Squire IB, et al. Healthcare needs and priorities of older people living with heart failure and frailty: a multi-perspective study of patients, carers and clinicians. BMC Geriatr. 2026;26:163. 10.1186/s12877-025-06926-1. PMC12870965 41495683

[7] Goyal P, Zainul O, Sharma Y, Reich A, Osma P, Lau JD, et al. Geriatric Vulnerabilities Among Adults With Heart Failure With Preserved Ejection Fraction. JACC: Advances. Am Coll Cardiol Foundation. 2025;4:101602. 10.1016/j.jacadv.2025.101602. PMC11994044 40155186

[8] Denfeld QE, Winters-Stone K, Mudd JO, Gelow JM, Kurdi S, Lee CS. The Prevalence of Frailty in Heart Failure: A Systematic Review and Meta-Analysis. Int J Cardiol. 2017;236:283–9. 10.1016/j.ijcard.2017.01.153. PMC5392144 28215466

[9] Jiang H, Wong JJ, Tan R-S, Gao F, Teo LLY, Strom JB, et al. Effect of Frailty on Cardiovascular Clinical Trials. JACC: Advances. Am Coll Cardiol Foundation. 2025;4:101889. 10.1016/j.jacadv.2025.101889. PMC12226365 40555009

[10] Swarbrick CJ, Williams K, Evans B, Blake HA, Poulton T, Nava S, et al. Postoperative outcomes in older patients living with frailty and multimorbidity in the UK: SNAP-3, a snapshot observational study. Br J Anaesth. 2025;135:166–76. 10.1016/j.bja.2025.04.026. PMC12226742 40425395

[11] Miles TJ, Ryan CT, Hogan KJ, Sayal BS, Sylvester CB, Rosengart TK, et al. Impact of frailty on outcomes and readmissions after transcatheter and surgical aortic valve replacement in a national cohort. JTCVS Open Elsevier. 2024;20:14–25. 10.1016/j.xjon.2024.05.006. PMC11405972 39296446

[12] Popat A, Vempati R, Kodali LSM, Sai Santhosha Mrudula A, Haddad F, Jain A, et al. Frailty status and outcomes of percutaneous coronary intervention in elderly patients with non-ST-elevation myocardial infarction. World J Cardiol. 2025;17:111870. 10.4330/wjc.v17.i10.111870. PMC12576566 41181652

[13] Rios S, Li W, Mustehsan MH, Hajra A, Takahashi T, Chengyue J, et al. Impact of Frailty on Outcomes After Transcatheter Edge-to-Edge Repair With MitraClip (from the National Inpatient Sample Database). Am J Cardiol. 2022;179:58–63. 10.1016/j.amjcard.2022.06.019. 35870989

[14] Ali S, Kumar M, Khlidj Y, Hendricks E, Farooq F, Alruwaili W, et al. Impact of frailty in hospitalized patients undergoing catheter ablation for atrial fibrillation. J Cardiovasc Electrophysiol. 2024;35:1929–38. 10.1111/jce.16383. 39075813

[15] Nakade T, Fujimoto Y, Singh YS, Akama Y, Ide T, Kida K, et al. Frailty Scale Captures Multidimensional Vulnerability and Predicts Mortality in Heart Failure. J Am Coll Cardiol. 2026;87:20–32. 10.1016/j.jacc.2025.09.1590. 41196240

[16] van Essen BJ, Emmens JE, Tromp J, Ouwerkerk W, Smit MD, Geluk CA, et al. Sex-specific risk factors for new-onset heart failure: the PREVEND study at 25 years. Eur Heart J. 2025;46:1528–36. 10.1093/eurheartj/ehae868. PMC12011521 39786471

[17] Castro C, Delwarde C, Shi Y, Roh J. Geroscience in heart failure: the search for therapeutic targets in the shared pathobiology of human aging and heart failure. J Cardiovasc Aging. 2025;5. 10.20517/jca.2024.15. PMC12036312 40297496

[18] Aman Y, Schmauck-Medina T, Hansen M, Morimoto RI, Simon AK, Bjedov I, et al. Autophagy in healthy aging and disease. Nat Aging Nat Publishing Group. 2021;1:634–50. 10.1038/s43587-021-00098-4. PMC8659158 34901876

[19] Raza S. Autophagy and metabolic aging: Current understanding and future applications. Biochimica et Biophysica Acta (BBA) - Mol Cell Research. 2024;1871:119753. 10.1016/j.bbamcr.2024.119753. 38763302

[20] Abdellatif M, Vasques-Nóvoa F, Trummer-Herbst V, Durand S, Koser F, Islam M, et al. Autophagy is required for the therapeutic effects of the NAD+ precursor nicotinamide in obesity-related heart failure with preserved ejection fraction. Eur Heart J. 2025;46:1863–6. 10.1093/eurheartj/ehaf062. PMC12075933 39995248

[21] Zhang H, Gao Y, Zhang M, Yuan Z, Chen Y, Wang A, et al. Schaftoside improves HFpEF through regulation the autophagy-lysosome pathway by allosterically targeting CaMKII-δ. Redox Biol. 2024;78:103424. 10.1016/j.redox.2024.103424. PMC11629582 39608246

[22] Rodrigues ACZ, Wang Z-M, Messi ML, Bonilla HJ, Liu L, Freeman WM, et al. Heart and neural crest derivative 2-induced preservation of sympathetic neurons attenuates sarcopenia with aging. Journal of Cachexia, Sarcopenia and Muscle. 2021;12:91–108. 10.1002/jcsm.12644. PMC7890150 33258279

[23] Chen Y, Maejima Y, Shirakabe A, Yamamoto T, Ikeda Y, Sadoshima J, et al. Ser9 phosphorylation of GSK-3β promotes aging in the heart through suppression of autophagy. J Cardiovasc Aging. 2021;1:9. 10.20517/jca.2021.13. PMC8589323 34778891

[24] Cui X, Spanos M, Zhao C, Wan W, Cui C, Wang L, et al. Mitochondrial dysfunction in HFpEF: potential interventions through exercise. J Cardiovasc Transl Res. 2025;18:442–56. 10.1007/s12265-025-10591-5. 39863753

[25] Chaanine AH, Joyce LD, Stulak JM, Maltais S, Joyce DL, Dearani JA, et al. Mitochondrial morphology, dynamics, and function in human pressure overload or ischemic heart disease with preserved or reduced ejection fraction. Circ Heart Fail. 2019;12:e005131. 10.1161/CIRCHEARTFAILURE.118.005131. 30744415

[26] Mortensen SA, Rosenfeldt F, Kumar A, Dolliner P, Filipiak KJ, Pella D, et al. The effect of coenzyme Q10 on morbidity and mortality in chronic heart failure: results from Q-SYMBIO: a randomized double-blind trial. JACC Heart Fail. 2014;2:641–9. 10.1016/j.jchf.2014.06.008. 25282031

[27] Butler J, Khan MS, Anker SD, Fonarow GC, Kim RJ, Nodari S, et al. Effects of elamipretide on left ventricular function in patients with heart failure with reduced ejection fraction: the PROGRESS-HF phase 2 trial. J Card Fail. 2020;26:429–37. 10.1016/j.cardfail.2020.02.001. 32068002

[28] Andreux PA, van Diemen MPJ, Heezen MR, Auwerx J, Rinsch C, Groeneveld GJ, et al. Mitochondrial function is impaired in the skeletal muscle of pre-frail elderly. Sci Rep. Nature Publishing Group; 2018;8:8548. 10.1038/s41598-018-26944-x. PMC5986740 29867098

[29] Alogna A, Koepp KE, Sabbah M, Espindola Netto JM, Jensen MD, Kirkland JL, et al. Interleukin-6 in patients with heart failure and preserved ejection fraction. JACC Heart Fail. 2023;11:1549–61. 10.1016/j.jchf.2023.06.031. PMC10895473 37565977

[30] Everett BM, Cornel JH, Lainscak M, Anker SD, Abbate A, Thuren T, et al. Anti-inflammatory therapy with canakinumab for the prevention of hospitalization for heart failure. Circulation. 2019;139:1289–99. 10.1161/CIRCULATIONAHA.118.038010. 30586730

[31] van Sleen Y, Shetty SA, van der Heiden M, Venema MCA, Gutiérrez-Melo N, Toonen EJM, et al. Frailty is related to serum inflammageing markers: results from the VITAL study. Immun Ageing. 2023;20:68. 10.1186/s12979-023-00391-3. PMC10680197 38012652

[32] Grootaert MOJ. Cell senescence in cardiometabolic diseases. npj Aging Nat Publishing Group. 2024;10:46. 10.1038/s41514-024-00170-4. PMC11493982 39433786

[33] Jia K, Dai Y, Liu A, Li X, Wu L, Lu L, et al. Senolytic agent navitoclax inhibits angiotensin II-induced heart failure in mice. J Cardiovasc Pharmacol. 2020;76:452–60. 10.1097/fjc.0000000000000878. 32675749

[34] Perez K, Ciotlos S, McGirr J, Limbad C, Doi R, Nederveen JP, et al. Single nuclei profiling identifies cell specific markers of skeletal muscle aging, frailty, and senescence. Aging (Albany NY). 2022;14:9393–422. 10.18632/aging.204435. PMC9792217 36516485

[35] Xu M, Pirtskhalava T, Farr JN, Weigand BM, Palmer AK, Weivoda MM, et al. Senolytics improve physical function and increase lifespan in old age. Nat Med. 2018;24:1246–56. 10.1038/s41591-018-0092-9. PMC6082705 29988130

[36] Cochran JD, Yura Y, Thel MC, Doviak H, Polizio AH, Arai Y, et al. Clonal Hematopoiesis in clinical and experimental heart failure with preserved ejection fraction. Circulation. 2023;148:1165–78. 10.1161/CIRCULATIONAHA.123.064170. PMC10575571 37681311

[37] Sano S, Oshima K, Wang Y, MacLauchlan S, Katanasaka Y, Sano M, et al. Tet2-Mediated Clonal Hematopoiesis Accelerates Heart Failure Through a Mechanism Involving the IL-1β/NLRP3 Inflammasome. J Am Coll Cardiol. 2018;71:875–86. 10.1016/j.jacc.2017.12.037. PMC5828038 29471939

[38] Jaiswal S, Fontanillas P, Flannick J, Manning A, Grauman PV, Mar BG, et al. Age-related clonal hematopoiesis associated with adverse outcomes. N Engl J Med. 2014;371:2488–98. 10.1056/NEJMoa1408617. PMC4306669 25426837

[39] Lipskerov F, Cohen-Kaplan V, Ciechanover A. Precise Mapping of the Proteasome Interaction Region (PIR) of p62/SQSTM1: Decoupling Condensate Formation from Proteasome Recruitment. Cells. 2026;15:335. 10.3390/cells15040335. PMC12939279 41744778

[40] Bektas A, Schurman SH, Candia J, Santiago-Fernández O, Kaushik S, Cuervo AM, et al. Preservation of Autophagy May Be a Mechanism Behind Healthy Aging. Aging Cell. 2025;24:e70246. 10.1111/acel.70246. PMC12611306 41078089

[41] Von Ruff ZD, Miller MJ, Moro T, Reidy PT, Ebert SM, Volpi E, et al. Resistance exercise training in older men reduces ATF4-activated and senescence-associated mRNAs in skeletal muscle. Geroscience. 2025;47:4601–22. 10.1007/s11357-025-01564-2. PMC12181168 40011348

[42] Barakat MF, Amaral N, Brayson D, Amin-Youssef G, Abu-Own H, Ayis S, et al. Ferric derisomaltose augments intrinsic skeletal muscle electron transport chain activity in heart failure: A FERRIC-HF II molecular substudy. Eur J Heart Fail. 2025;27:2343–51. 10.1002/ejhf.70028. PMC12765364 40916716

[43] Raffa S, Forte M, Gallo G, Ranieri D, Marchitti S, Magrì D, et al. Atrial natriuretic peptide stimulates autophagy/mitophagy and improves mitochondrial function in chronic heart failure. Cell Mol Life Sci. 2023;80:134. 10.1007/s00018-023-04777-w. PMC10133375 37099206

[44] Katsuumi G, Shimizu I, Suda M, Yoshida Y, Furihata T, Joki Y, et al. SGLT2 inhibition eliminates senescent cells and alleviates pathological aging. Nat Aging. 2024;4:926–38. 10.1038/s43587-024-00642-y. PMC11257941 38816549

[45] Hu X, Li D, Chen W, Kuang H, Yang D, Gong Z, et al. Sodium Glucose Transporter 2 Inhibitor Protects Against Heart Failure With Preserved Ejection Fraction: Preclinical ‘2-Hit’ Model Reveals Autophagy Enhancement Via AMP-Activated Protein Kinase/Mammalian Target of Rapamycin Complex 1 Pathway. J Am Heart Assoc. 2025;14:e040093. 10.1161/JAHA.124.040093. PMC12533654 40767292

[46] Verma S, Petrie MC, Borlaug BA, Butler J, Davies MJ, Kitzman DW, et al. Inflammation in Obesity-Related HFpEF: The STEP-HFpEF Program. J Am Coll Cardiol. 2024;84:1646–62. 10.1016/j.jacc.2024.08.028. 39217564

[47] Borlaug BA, Zile MR, Kramer CM, Baum SJ, Hurt K, Litwin SE, et al. Effects of tirzepatide on circulatory overload and end-organ damage in heart failure with preserved ejection fraction and obesity: a secondary analysis of the SUMMIT trial. Nat Med. 2025;31:544–51. 10.1038/s41591-024-03374-z. PMC11835708 39551891

[48] Haykowsky MJ, Tomczak CR, Scott JM, Paterson DI, Kitzman DW. Determinants of exercise intolerance in patients with heart failure and reduced or preserved ejection fraction. J Appl Physiol (1985). 2015;119:739–44. 10.1152/japplphysiol.00049.2015. PMC4687865 25911681

[49] Cicoira M, Zanolla L, Franceschini L, Rossi A, Golia G, Zamboni M, et al. Skeletal muscle mass independently predicts peak oxygen consumption and ventilatory response during exercise in noncachectic patients with chronic heart failure. J Am Coll Cardiol. 2001;37:2080–5. 10.1016/s0735-1097(01)01306-7. 11419891

[50] Damluji AA, Alfaraidhy M, AlHajri N, Rohant NN, Kumar M, Al Malouf C, et al. Sarcopenia and Cardiovascular Diseases. Circulation Am Heart Association. 2023;147:1534–53. 10.1161/CIRCULATIONAHA.123.064071. PMC10180053 37186680

[51] Kedlian VR, Wang Y, Liu T, Chen X, Bolt L, Tudor C, et al. Human skeletal muscle aging atlas. Nat Aging Nat Publishing Group. 2024;4:727–44. 10.1038/s43587-024-00613-3. PMC11108788 38622407

[52] Lavine KJ, Sierra OL. Skeletal muscle inflammation and atrophy in heart failure. Heart Fail Rev. 2017;22:179–89. 10.1007/s10741-016-9593-0. PMC5352459 28091823

[53] Grevendonk L, Connell NJ, McCrum C, Fealy CE, Bilet L, Bruls YMH, et al. Impact of aging and exercise on skeletal muscle mitochondrial capacity, energy metabolism, and physical function. Nat Commun Nat Publishing Group. 2021;12:4773. 10.1038/s41467-021-24956-2. PMC8346468 34362885

[54] Ramírez-Vélez R, González A, García-Hermoso A, Amézqueta IL, Izquierdo M, Díez J. Revisiting skeletal myopathy and exercise training in heart failure: Emerging role of myokines. Metabolism. 2023;138:155348. 10.1016/j.metabol.2022.155348. 36410495

[55] Lecker SH, Zavin A, Cao P, Arena R, Allsup K, Daniels KM, et al. Expression of the Irisin Precursor FNDC5 in Skeletal Muscle Correlates With Aerobic Exercise Performance in Patients With Heart Failure. Circ Heart Fail. 2012;5:812–8. 10.1161/CIRCHEARTFAILURE.112.969543. PMC7284011 23001918

[56] Chen Y, Qian M, Gao F, Li G, Peng K, Sun Q, et al. Potential effect of Irisin on sarcopenia: a systematic review. BMC Musculoskelet Disord. 2025;26:520. 10.1186/s12891-025-08767-w. PMC12107908 40420047

[57] Baek JY, Jang I-Y, Jung H-W, Park SJ, Lee JY, Choi E, et al. Serum irisin level is independent of sarcopenia and related muscle parameters in older adults. Exp Gerontol. 2022;162:111744. 10.1016/j.exger.2022.111744. 35182609

[58] Lourenco MV, Frozza RL, de Freitas GB, Zhang H, Kincheski GC, Ribeiro FC, et al. Exercise-linked FNDC5/irisin rescues synaptic plasticity and memory defects in Alzheimer’s models. Nat Med. 2019;25:165–75. 10.1038/s41591-018-0275-4. PMC6327967 30617325

[59] Roh E, Hwang SY, Song E, Park MJ, Yoo HJ, Baik SH, et al. Association of plasma brain-derived neurotrophic factor levels and frailty in community-dwelling older adults. Sci Rep. 2022;12:18605. 10.1038/s41598-022-19706-3. PMC9633836 36329115

[60] Fukushima A, Kinugawa S, Homma T, Masaki Y, Furihata T, Yokota T, et al. Decreased serum brain-derived neurotrophic factor levels are correlated with exercise intolerance in patients with heart failure. Int J Cardiol. 2013;168:e142–4. 10.1016/j.ijcard.2013.08.073. 24029660

[61] Edman S, Horwath O, Van der Stede T, Blackwood SJ, Moberg I, Strömlind H, et al. Pro-Brain-Derived Neurotrophic Factor (BDNF), but Not Mature BDNF, Is Expressed in Human Skeletal Muscle: Implications for Exercise-Induced Neuroplasticity. Function (Oxf). 2024;5:zqae005. 10.1093/function/zqae005. PMC11065112 38706964

[62] Behnoush AH, Khalaji A, Fazlollahpour-Naghibi A, Bagheri K, Goshtasbi P, Mohseni G, et al. Circulating brain‐derived neurotrophic factor levels and heart failure: A systematic review and meta‐analysis. ESC Heart Fail. 2024;11:3253–63. 10.1002/ehf2.14916. PMC11424347 38923432

[63] Pisani A, Paciello F, Del Vecchio V, Malesci R, De Corso E, Cantone E, et al. The Role of BDNF as a Biomarker in Cognitive and Sensory Neurodegeneration. J Personalized Med Multidisciplinary Digit Publishing Inst. 2023;13:652. 10.3390/jpm13040652. PMC10140880 37109038

[64] Vinel C, Lukjanenko L, Batut A, Deleruyelle S, Pradère J-P, Le Gonidec S, et al. The exerkine apelin reverses age-associated sarcopenia. Nat Med Nat Publishing Group. 2018;24:1360–71. 10.1038/s41591-018-0131-6. 30061698

[65] Pitkin SL, Maguire JJ, Kuc RE, Davenport AP. Modulation of the apelin/APJ system in heart failure and atherosclerosis in man. Br J Pharmacol. 2010;160:1785–95. 10.1111/j.1476-5381.2010.00821.x. PMC2936849 20649580

[66] Sato T, Kadowaki A, Suzuki T, Ito H, Watanabe H, Imai Y, et al. Loss of Apelin Augments Angiotensin II-Induced Cardiac Dysfunction and Pathological Remodeling. Int J Mol Sci. 2019;20:239. 10.3390/ijms20020239. PMC6358887 30634441

[67] Berry MF, Pirolli TJ, Jayasankar V, Burdick J, Morine KJ, Gardner TJ, et al. Apelin has in vivo inotropic effects on normal and failing hearts. Circulation. 2004;110:II187–193. 10.1161/01.CIR.0000138382.57325.5c. 15364861

[68] Kistner TM, Tavormina A, Lieberman DE. Myokine secretion during moderate-intensity physical activity: Dose-response of interleukin 6 to walking duration. Am J Hum Biol. 2024;36:e24131. 10.1002/ajhb.24131. 39030918

[69] Mooney L, Jackson CE, Adamson C, McConnachie A, Welsh P, Myles RC, et al. Adverse Outcomes Associated With Interleukin-6 in Patients Recently Hospitalized for Heart Failure With Preserved Ejection Fraction. Circ Heart Fail. 2023;16:e010051. 10.1161/CIRCHEARTFAILURE.122.010051. PMC10101136 36896709

[70] Lenk K, Erbs S, Höllriegel R, Beck E, Linke A, Gielen S, et al. Exercise training leads to a reduction of elevated myostatin levels in patients with chronic heart failure. Eur J Prev Cardiol. 2012;19:404–11. 10.1177/1741826711402735. 21450574

[71] Chen P, Liu Z, Luo Y, Chen L, Li S, Pan Y, et al. Predictive value of serum myostatin for the severity and clinical outcome of heart failure. Eur J Intern Med. 2019;64:33–40. 10.1016/j.ejim.2019.04.017. 31056368

[72] Chu R, Li M, Zhu C, Du Y, Xie Y, Ni T, et al. The effects of resistance training on myostatin in older adults with frailty and/or sarcopenia: a systematic review and meta analysis. BMC Sports Sci Med Rehabil. 2026;18:222. 10.1186/s13102-026-01655-3. PMC13141354 41888949

[73] Wang Q, Wang H, Tian H, Niu S, Xu R. A Prospective Case-Control Study Examining the Relationship Between Frailty and Serum Myostatin in Older Persons with Chronic Heart Failure. Risk Manag Healthc Policy. 2023;16:1343–9. 10.2147/RMHP.S412725. PMC10365999 37497257

[74] Szaroszyk M, Kattih B, Martin-Garrido A, Trogisch FA, Dittrich GM, Grund A, et al. Skeletal muscle derived Musclin protects the heart during pathological overload. Nat Commun Nat Publishing Group. 2022;13:149. 10.1038/s41467-021-27634-5. PMC8748430 35013221

[75] Subbotina E, Sierra A, Zhu Z, Gao Z, Koganti SRK, Reyes S, et al. Musclin is an activity-stimulated myokine that enhances physical endurance. Proc Natl Acad Sci U S A. 2015;112:16042–7. 10.1073/pnas.1514250112. PMC4702977 26668395

[76] Kattih B, Carstens DC, Boeckling F, Rasper T, Pergola G, Dimmeler S, et al. Low Circulating Musclin is Associated With Adverse Prognosis in Patients Undergoing Transcatheter Aortic Valve Implantation at Low-Intermediate Risk. J Am Heart Assoc. 2022;11:e022792. 10.1161/JAHA.121.022792. PMC9075297 35229655

[77] Køber L, Adamo M, Ruwald A-C, Tomasoni D, Anderson LJ, Andersson C, et al. 2026 ESC Guidelines for the management of heart failure. Eur Heart J. 2026;47:5346–457. 10.1093/eurheartj/ehag100. 42661420

[78] Vitale C, Jankowska E, Hill L, Piepoli M, Doehner W, Anker SD, et al. Heart Failure Association of the European Society of Cardiology position paper on frailty in patients with heart failure. Eur J Heart Fail. 2019;21:1299–305. 10.1002/ejhf.1611. 31646718

[79] Heidenreich PA, Bozkurt B, Aguilar D, Allen LA, Byun JJ, Colvin MM, et al. 2022 AHA/ACC/HFSA Guideline for the Management of Heart Failure: A Report of the American College of Cardiology/American Heart Association Joint Committee on Clinical Practice Guidelines. Circulation Am Heart Association. 2022;145:e895–1032. 10.1161/CIR.0000000000001063. 35363499

[80] Andersson ÅG, Dahlkvist L, Kurland L. Patient-centered outcomes and outcome measurements for people aged 65 years and older—a scoping review. BMC Geriatr. 2024;24:528. 10.1186/s12877-024-05134-7. PMC11186133 38890618

[81] Akpan A, Roberts C, Bandeen-Roche K, Batty B, Bausewein C, Bell D, et al. Standard set of health outcome measures for older persons. BMC Geriatr. 2018;18:36. 10.1186/s12877-017-0701-3. PMC5797357 29394887

[82] Prorok JC, Williamson PR, Shea B, Rolfson D, Mañas LR, Cesari M, et al. An international Delphi consensus process to determine a common data element and core outcome set for frailty: FOCUS (The Frailty Outcomes Consensus Project). BMC Geriatr. 2022;22:284. 10.1186/s12877-022-02993-w. PMC8985339 35382752

[83] Gouda P, Rathwell S, Colin-Ramirez E, Felker GM, Ross H, Escobedo J, et al. Utilizing Quality of Life Adjusted Days Alive and Out of Hospital in Heart Failure Clinical Trials. Circ Cardiovasc Qual Outcomes. 2024;17:e010560. 10.1161/CIRCOUTCOMES.123.010560. 38567506

[84] Butt JH, Dewan P, Merkely B, Belohlávek J, Drożdż J, Kitakaze M, et al. Efficacy and Safety of Dapagliflozin According to Frailty in Heart Failure With Reduced Ejection Fraction: A Post Hoc Analysis of the DAPA-HF Trial. Ann Intern Med. 2022;175:820–30. 10.7326/M21-4776. 35467935

[85] Raijmann RCMA, van Dalen TN, Koek HL, van der Meer MG, Emmelot-Vonk MH, Keijsers CJPW. Effect of geriatric care on health outcomes in older patients with cardiac disease: A systematic review. Arch Gerontol Geriatr. 2025;132:105786. 10.1016/j.archger.2025.105786. 39985873

[86] Van Grootven B, Jeuris A, Jonckers M, Devriendt E, Dierckx de Casterlé B, Dubois C, et al. Geriatric co-management for cardiology patients in the hospital: A quasi-experimental study. J Am Geriatr Soc. 2021;69:1377–87. 10.1111/jgs.17093. 33730373

[87] Raijmann RCMA, Koek HL, Emmelot-Vonk MH, Swaving JGE, Agema WRP, Kerckhoffs APM, et al. Impact of geriatric co-management on outcomes in hospitalised cardiology patients aged 85 and over. Neth Heart J. 2024;32:76–83. 10.1007/s12471-023-01806-y. PMC10834903 37651030

[88] Herrero-Torrus M, Badosa N, Roqueta C, Ruiz-Bustillo S, Solé-González E, Belarte-Tornero LC, et al. Randomized Controlled Trial Comparing a Multidisciplinary Intervention by a Geriatrician and a Cardiologist to Usual Care after a Heart Failure Hospitalization in Older Patients: The SENECOR Study. Journal of Clinical Medicine. Multidisciplinary Digit Publishing Inst. 2022;11:1932. 10.3390/jcm11071932. PMC8999953 35407540

[89] Okoye C, Mazzarone T, Finazzi A, Daniela G, Bruni AA, Maccioni L, et al. Outcomes of early post-discharge cardio-geriatric care in frail patients after acute heart failure: a before-and-after study. BMC Geriatr. 2025;25:236. 10.1186/s12877-025-05883-z. PMC11983887 40205565

[90] Kitzman DW, Whellan DJ, Duncan P, Pastva AM, Mentz RJ, Reeves GR, et al. Physical Rehabilitation for Older Patients Hospitalized for Heart Failure. New Engl J Med Mass Med Soc. 2021;385:203–16. 10.1056/NEJMoa2026141. PMC8353658 33999544

[91] Bonilla-Palomas JL, Gámez-López AL, Castillo-Domínguez JC, Moreno-Conde M, López Ibáñez MC, Alhambra Expósito R, et al. Nutritional Intervention in Malnourished Hospitalized Patients with Heart Failure. Arch Med Res. 2016;47:535–40. 10.1016/j.arcmed.2016.11.005. 28262195

[92] Pastva AM, Reeves GR, Whellan DJ, Mentz RJ, Chen H, Bertoni AG, et al. Physical rehabilitation for older patients with acute HFpEF (REHAB-HFpEF) trial: Design and rationale. Am Heart J. 2026;297:107420. 10.1016/j.ahj.2026.107420. PMC13077654 41812966

[93] The CHART trial. – comprehensive assessment for older people with heart failure and frailty [Internet]. ISRCTN Registry. [cited 2026 July 7]. 10.1186/ISRCTN22229432

[94] Kondo T, Adachi T, Kobayashi K, Okumura T, Izawa H, Murohara T, et al. Physical Frailty and Use of Guideline-Recommended Drugs in Patients With Heart Failure and Reduced Ejection Fraction. J Am Heart Association Wiley. 2023;12:e026844. 10.1161/JAHA.122.026844. PMC10356033 37301739

[95] Butt JH, Jhund PS, Henderson AD, Claggett BL, Chiang C-E, Linssen GCM, et al. Finerenone According to Frailty in Heart Failure: A Prespecified Analysis of the FINEARTS-HF Randomized Clinical Trial. JAMA Cardiol. 2025;10:829–40. 10.1001/jamacardio.2025.1775. PMC12177723 40531488

[96] Cohen-Solal A, McMurray JJV, Swedberg K, Pfeffer MA, Puu M, Solomon SD, et al. Benefits and safety of candesartan treatment in heart failure are independent of age: insights from the Candesartan in Heart failure–Assessment of Reduction in Mortality and morbidity programme. Eur Heart J. 2008;29:3022–8. 10.1093/eurheartj/ehn476. 18987098

[97] Kotecha D, Manzano L, Krum H, Rosano G, Holmes J, Altman DG, et al. Effect of age and sex on efficacy and tolerability of β blockers in patients with heart failure with reduced ejection fraction: individual patient data meta-analysis. BMJ. 2016;353:i1855. 10.1136/bmj.i1855. PMC4849174 27098105

[98] Kobayashi M, Yamashina A, Satomi K, Tezuka A, Ito S, Asakura M, et al. Adverse events associated with early initiation of Eplerenone in patients hospitalized for acute heart failure. Int J Cardiol. 2024;415:132477. 10.1016/j.ijcard.2024.132477. 39181408

[99] Hacil A, Antakly Hanon Y, Lacour A, David J-P, Khalifa T, Piccoli M et al. Efficacy and Safety of SGLT2 Inhibitors in Heart Failure: Observational Evidence in Geriatric Patients AGING-HF. Circulation: Heart Failure. Am Heart Association; 0:e012794. 10.1161/CIRCHEARTFAILURE.125.012794 40832702

[100] Mebazaa A, Davison B, Chioncel O, Cohen-Solal A, Diaz R, Filippatos G et al. Safety, tolerability and efficacy of up-titration of guideline-directed medical therapies for acute heart failure (STRONG-HF): a multinational, open-label, randomised, trial. Lancet. London, England; 2022;400:1938–52. 10.1016/S0140-6736(22)02076-1 36356631

